# Prenatal methotrexate injection increases behaviors possibly associated with depression and/or autism in rat offspring; A new animal model for mental disorder, based on folate metabolism deficit during pregnancy

**DOI:** 10.1002/npr2.12255

**Published:** 2022-05-03

**Authors:** Naoki Amada, Yusuke Kakumoto, Takashi Futamura, Kenji Maeda

**Affiliations:** ^1^ Department of CNS Research Otsuka Pharmaceutical Co., Ltd. Tokushima Japan; ^2^ Department of Lead Discovery Research Otsuka Pharmaceutical Co., Ltd. Tokushima Japan

**Keywords:** autism, depression, folate, methotrexate, psychiatric disorders

## Abstract

**Background:**

Deficiency of folate, an essential vitamin for DNA synthesis and methylation, is reported as a risk factor for mental disorders. Considering a possibility that folate metabolism deficit during pregnancy may disturb CNS development and increase mental disorders in offspring, we treated pregnant rats with methotrexate (MTX), an inhibitor of folate metabolic enzyme, and evaluated offspring behaviors.

**Methods:**

Saline or MTX was intraperitoneally administered to female SD rats on gestational day 17. Offspring behaviors were evaluated during approximately 6–9 weeks old; prepulse inhibition (PPI), social interaction (SI), locomotor activity (LA), and forced swimming test (FST) for evaluation of schizophrenia, depression, and autism related behaviors; the elevated plus maze (EPM) and the light–dark box (LD) test for evaluation of anxiety.

**Results:**

Compared to saline‐treated group, MTX‐treated group showed decrease of SI and increase of immobility time in FST. In addition, increases of time spent in the light box and shuttling between the light–dark boxes were observed in LD test. On the other hand, no changes were confirmed in EPM, LA, and PPI.

**Conclusion:**

Decrease of SI and increase of immobility time in FST may suggest association of this animal model with depression and/or autism. Increase of time spent in the light box and shuttling between the light–dark boxes may indicate changes in anxiety or cognitive level to environment, or repetitive behaviors in autism. Although further studies are warranted to characterize this animal model, at least we can say that prenatal MTX exposure, possibly causing folate metabolism deficit, affects offspring behaviors.

## 1 INTRODUCTION

Chemically induced acute animal models (eg, hyperlocomotion and impairment of prepulse inhibition induced by MK‐801, amphetamine, and etc) are often used for evaluation/selection of novel compounds for treatment of psychiatric disorders^1^, ^2^, ^3^ because these are high‐throughput screening models. However, these are mostly animal models reflecting a facet (symptom) of a disease and behavioral changes are normally acute. Therefore, disease models, spontaneously exhibiting multifaceted phenotypes of a disease, are needed especially for evaluation of disease‐modifying drugs as well as pathological investigation of psychiatric disorders. For this reason, we tried to establish a novel animal model of psychiatric disorder.

It has been known that nutritional deficiencies in early gestation due to exposure to famines in Netherlands and China in the mid‐20th century increased a risk of schizophrenia, approximately 2‐fold, in offspring.^4^ Folate was suggested to be one of candidate micronutrients lost during the famines, which increased the risk of schizophrenia. Indeed, maternal folate deficiency and hyperhomocystinemia are suggested as potential prenatal risk factors of schizophrenia.^5^ Moreover, meta‐analyses revealed decreased serum/plasma levels of folate in patients with schizophrenia.^6^, ^7^ Folate concentration was inversely correlated with the Scale for Assessment of Negative Symptom total score in schizophrenic patients.^8^ Supplementation of folate and vitamin B12 improved negative symptoms of schizophrenia.^9^ To date, aberrant blood levels of folate, homocysteine, and vitamin B12 have been reported not only in schizophrenia, but also in other psychiatric disorders such as major depressive disorder (MDD), bipolar disorder, and obsessive compulsive disorder.^10^, ^11^, ^12^, ^13^ In addition, urinary metabolite analysis revealed changes associated with folate and vitamin B12 deficiency and homocysteine accumulation in children with autism spectrum disorder (ASD).^14^ It has been reported that folate receptor alpha autoantibodies were found in patients with schizophrenia^15^ and also in large part of children with ASD.^16^ Therefore, folate deficiency and/or insufficient folate metabolism seem to play an important role in etiology of psychiatric disorders such as schizophrenia.

Folate is a crucial one carbon source in cellular pathways for DNA, RNA, and protein methylation, and DNA synthesis, in which folate is metabolized by more than 30 enzymes including dihydrofolate reductase (DHFR) and methylenetetrahydrofolate reductase (MTHFR).^17^, ^18^, ^19^ For instance, DHFR catalyzes the reduction of dietary folate and dihydrofolate to tetrahydrofolate. Tetrahydrofolate is then converted to 5,10‐methylenetetrahydrofolate by thymidylate synthase, which is an important donor for methyl group in DNA synthesis and DNA methylation.^18^ MTHFR catalyzes conversion of 5,10‐methylenetetrahydrofolate to 5‐methyltetrahydrofolate. Then, methionine synthase catalyzes transfer of a methyl group from 5‐methyltetrahydrofolate to homocysteine to generate methionine for DNA methylation, in which vitamin B12 acts as a cofactor.^18^, ^20^, ^21^ DNA methylation is important epigenetic determinant in gene expression, DNA stability, and DNA integrity.^18^

Folate deficiency during pregnancy is known as a causative factor for neural tube defects, and folate supplementation prevents the risk.^22^, ^23^ It has been reported that folate promotes neural stem cell proliferation and neurogenesis.^24^, ^25^ These indicate that folate deficiency and/or folate metabolism deficit are likely to disturb normal development of nervous system and neural networks.

Taken together, there is a possibility that folate deficiency and/or insufficient folate metabolism during pregnancy decreases neural stem cell proliferation and differentiation in embryos, subsequently this in turn disturbs normal central nervous system (CNS) development and may increase susceptibility to mental disorders such as schizophrenia in offspring. Based on this theory, in this study, we tried to generate a new animal model of schizophrenia for which we treated methotrexate (MTX), a DHFR inhibitor, to pregnant rats to inhibit folate metabolism and evaluated offspring behaviors associated with schizophrenia (prepulse inhibition, social interaction, and locomotor activity; as primary outcomes in this study). In addition, we conducted other behavioral tests for exploratory purpose (forced swim test, elevated plus maze, and light–dark test; as secondary outcomes).

## 2 METHODS

### 2.1 Animals and treatment

**FIGURE 1:**
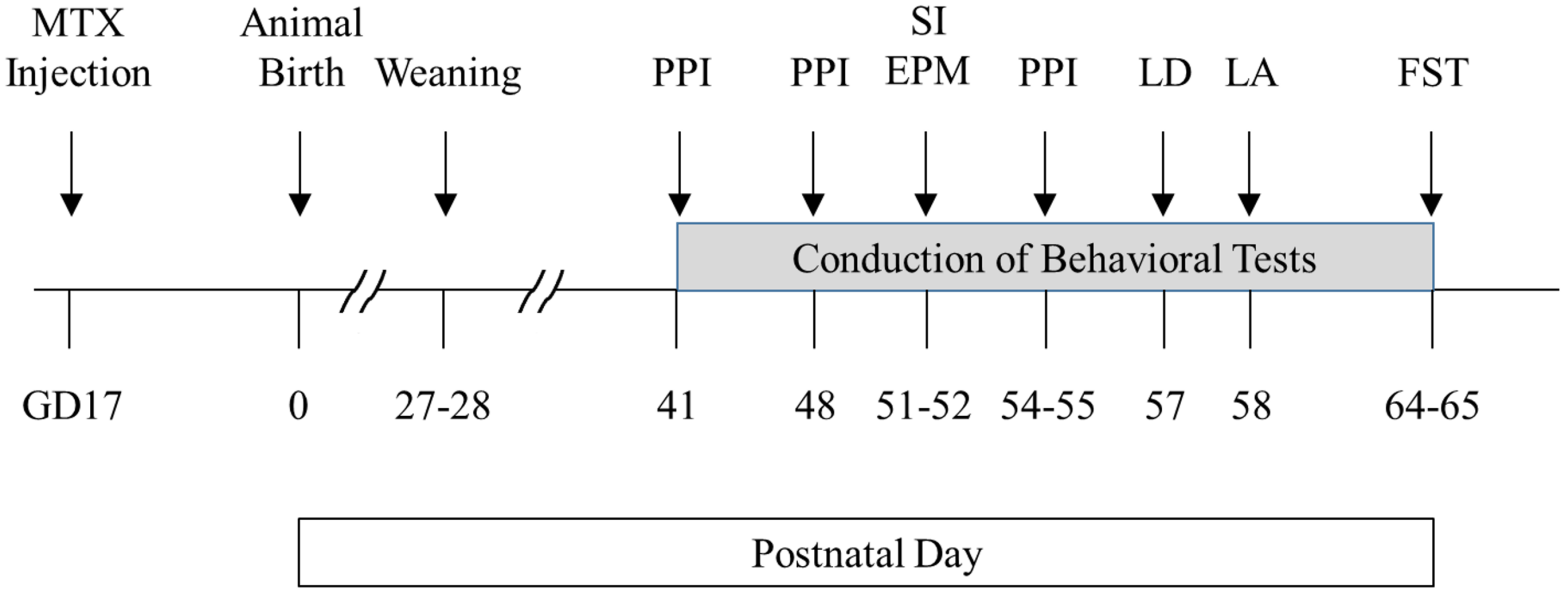
Schematic diagram of experimental procedures. Abbreviations; MTX: methotrexate, GD: gestational day, PPI: prepulse inhibition, SI: social interaction, EPM: the elevated plus maze, LD: the light–dark box test, LA: locomotor activity, FST: the forced swimming test. Female rats were i.p. injected with saline or MTX (5 mg/kg) on GD17. Offspring rats were weaned between postnatal day (PD) 27 and 28. PPI was measured on PD41, PD48, and PD54 (or 55). SI, EPM, LD, and LA were conducted on PD51, 52, 57, and 58, respectively. FST was conducted on PD64 (training session) and PD65 (test session). *Note*: Albeit not tested in a blinded manner, in order to perform this study at high quality, MTX injection, animal care, and behavioral assessments were conducted by two well skilled experimenters, in which each behavioral experiment was run by either of the 2 persons

Six female pregnant Sprague–Dawley rats (Japan SLC, Inc., Japan) were used. Two rats were intraperitoneally (i.p.) injected with saline and 4 rats were i.p. injected with MTX (5 mg/kg; Takeda Pharmaceutical Co., Ltd., Japan) on gestational day (GD) 17 (Figure 1). The reason why the injection timing was decided is that neurogenesis in rat medulla and spinal cord, those regions necessary for basic bio‐functions such as respiration, nearly finishes by GD17, but still neurogenesis in other brain regions including neocortex and hippocampus is occurring on GD17, and also neuronal differentiation and migration are ongoing.^26^ Therefore, MTX exposure on GD17 would affect neuronal development in the forebrain.

Pups of one of MTX‐treated rats were found dead after birth. All other offspring were alive and used for behavioral evaluation. Offspring rats were weaned at approximately 4 weeks old and started housing female and male separately from 5 weeks old. Thereafter, offspring behaviors were evaluated during approximately 6–9 weeks old (Figure 1). Animals were group housed in cages with water and food (Oriental Yeast Co, Ltd) supplied ad libitum, and maintained under artificial lighting between 7:00 am to 7:00 pm. The room temperature and humidity were maintained at 23 ± 2 °C and 60 ± 10%, respectively.

### 2.2 Prepulse inhibition

Measurement of acoustic startle response and prepulse inhibition (PPI) was conducted according to Futamura et al.^27^ Acoustic startle response was measured in a startle chamber (SR‐Lab Systems, San Diego Instruments, San Diego, CA, USA) adapted for rats. Acoustic stimuli of 120 dB, a single prepulse interval (100 ms), and three different prepulse intensities (5, 10, and 15 dB above background noise [white noise, 70 dB]) were used. Each rat was placed in the startle chamber and initially acclimatized for 5 minutes with background noise alone. The rat was then subjected to 40 startle trials, each trial consisting of one of five conditions; (i) a 40‐ms 120‐dB noise burst presented alone; (ii)–(iv) a 40‐ms 120‐dB noise burst following prepulses by 100 ms (20‐ms noise burst) that were 5, 10, or 15 dB above background noise (ie, 75‐, 80‐, or 85‐dB prepulse, respectively); or (v) no stimulus (background noise alone), which was used to measure baseline movement in the chamber. These five trial types (i)–(v) were each repeated eight times in a pseudorandom order to give 40 trials. The intertrial interval was 15 s. Each trial type was presented once within a block of five trials. Analysis was based on the mean of the seven trials (ignoring the first trial) for each trial type. The percentage PPI of a startle response was calculated as: 100 − [(startle response on prepulse–pulse stimulus trials−no stimulus trials)/(pulse‐alone trials−no stimulus trials)] × 100).

### 2.3 Social interaction

The index for social interaction (SI) of rodents was measured according to Futamura et al.^27^ In brief, each test rat was placed in an open field box for 10 minutes with a stranger rat (partner) that was housed in a different cage, and was age, body weight, and gender‐matched. Testing was performed under high light level (1200 lux) and unfamiliar condition. Duration of SI was measured based on sniffing behaviors, defined as active chasing of the partner, shaking the nose near the partner, and contacting the partner with the nose.

### 2.4 Elevated plus maze

The apparatus of the elevated plus maze (EPM; Yamashita Giken Co., Tokushima, Japan) consisted of two opposite open arms without walls (50 cm × 10 cm) and two enclosed arms (closed arm: 50 cm × 10 cm) with 25 cm height side and end walls, extending from a platform of central square (10 cm × 10 cm). The entire apparatus was elevated 50 cm above the floor. The EPM test was conducted in a dimly room and the apparatus was lit by two stand lights. At the start of each trial, each rat was placed at the center of maze, facing toward one of closed arms. Time spent in open arms and entries to open and closed arms were counted for 5 minutes per animal.

### 2.5 Light dark box test

Each rat was placed in the dark box of a light–dark (LD) test apparatus (Yamashita Giken Co., Tokushima, Japan). The light box was lit at 400 lux. Time spent in the light box, and entries to the light box and the dark box were counted for 5 minutes per animal. When a rat put all four limbs onto the floor of the light or dark box, it was defined as animal entry to the box.

### 2.6 Locomotor activity

Locomotor activity (LA) was measured using a LA meter (Yamashita Giken Co., Tokushima, Japan) with a method described by Mori et al.^28^ Each rat was placed in a plastic circular chamber (30 cm diameter and 30 cm height) and LA was measured for 30 minutes. In the LA meter, a pivot was fixed at the center of each chamber, where the animal was placed, and six micro‐switches were fitted beneath the perimeter. Locomotor movement of the animals generated a pulse by switching the micro‐switch and electrically charging a magnetic counter.

### 2.7 Forced swimming test

The forced swimming test (FST) was conducted, with slight modification, according to Tottori et al.^29^ To induce conditioned immobility (training session), rats were placed in a clear plastic cylinder (17 cm diameter and 40 cm height) filled with water maintained at room temperature for 5 minutes on the day before the test. On the testing day (test session), each rat was placed in the water again and swimming time was measured for 5 minutes using an auto‐activity analysis system with infrared rays (SCANET MV‐10 AQ, Toyo Sangyo Co., Ltd., Japan).

### 2.8 Statistical analysis

The values of the behavioral test results were expressed as mean ± SEM. All statistical analyses were performed using SAS Software for Windows, Release 9.4 (SAS Institute Japan Ltd.). The differences were considered statistically significant, when *P* value was <0.05.

For PPI, SI, LA, EPM, and LD tests, data were analyzed for the overall measurement time of each test, and the differences in individual parameters were analyzed between saline‐treated group and MTX‐treated group using a two‐tailed t‐test.

For FST, swimming time (mobility time) was tallied per minute (0–1, 1–2, 2–3, 3–4, and 4–5 minute) and the data were compared between saline‐treated group and MTX‐treated group over the time course (0 to 5 minutes) by a mixed‐effect model for repeated measures (MMRM) method using compound symmetric variance covariance structure. Once confirming there was a significant difference in the main effect (group difference over the time course), an unpaired t‐test with a time‐by‐drug interaction was conducted on swimming time at each time point using a closed test procedure (procedure to control the experimentwise or multiple type I error rate when multiple hypotheses have to be tested).

The difference in total immobility time for the overall measurement time (5 minutes) was analyzed by a two‐tailed t‐test.

Weekly body weights were compared between the two groups using the same procedures mentioned for swimming time (the MMRM method and the closed testing procedure).

Considering that multiple adjustment is applied to primary outcomes,^30^ *P*‐values of the three primary outcomes (PPI, SI, and LA) were adjusted by Bonferroni correction.

## 3 RESULTS

**FIGURE 2:**
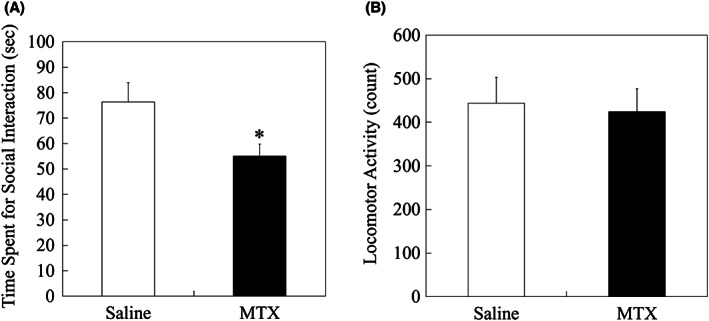
Results of social interaction and locomotor activity. (A) Social interaction (SI) result. Significantly less SI time was confirmed in MTX group compared to saline group, analyzed by a two‐tailed t‐test (*: *P* < 0.05). (B) Locomotor activity (LA) result. No significant LA difference was found between saline and MTX groups, analyzed by a two‐tailed t‐test. Data are expressed as mean ± SEM. The number of animals was n = 21 and n = 26 for saline group and MTX group, respectively

MTX‐treated group showed significantly less SI time compared to saline‐treated group (p = 0.0495, adjusted by Bonferonni correction; Figure 2A), indicating less sociability in MTX‐treated animals. Despite the SI reduction in MTX‐treated group, no significant difference was found in LA between saline‐group and MTX‐treated group (*P* = 1.0000, adjusted by Bonferonni correction; Figure 2B), indicating that the SI reduction in MTX‐treated group was not caused by animal activity change.

**FIGURE 3:**
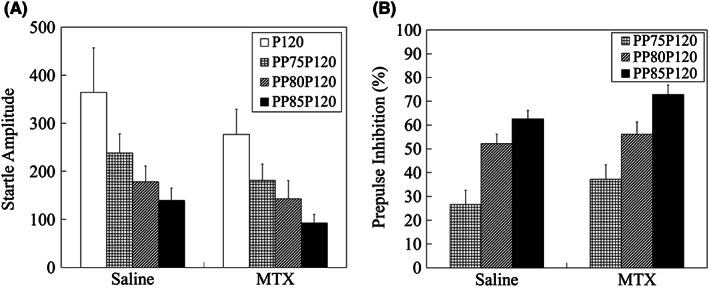
Results of prepulse inhibition test. (A) Startle amplitude. (B) Prepulse inhibition (PPI). There were no significant differences in startle amplitude and PPI between saline group and MTX group, analyzed by a two‐tailed t‐test. Data are expressed as mean ± SEM. The number of animals was n = 21 and n = 26 for saline group and MTX group, respectively. P120: Pulse 120 dB, PP75: Prepulse 75 dB, PP80: Prepulse 80 dB, PP85: Prepulse 85 dB

There were no clear differences in startle amplitude (SA) and PPI between saline‐treated group and MTX‐treated group on all test days. Considering data from PD41, PD48, and PD54 (or 55) were all similar, only the results from PD48 are shown as data example in Figure 3 (SA at 120 dB pulse: *P* = 1.0000, SA at 75 dB prepulse +120 dB pulse: *P* = 0.8334, SA at 80 dB prepulse +120 dB pulse: *P* = 1.0000, SA at 85 dB prepulse +120 dB pulse: *P* = 0.3777, PPI by 75 dB prepulse: *P* = 0.6597, PPI by 80 dB prepulse: *P* = 1.0000, PPI by 85 dB prepulse: *P* = 0.1977, adjusted by Bonferonni correction).

**FIGURE 4:**
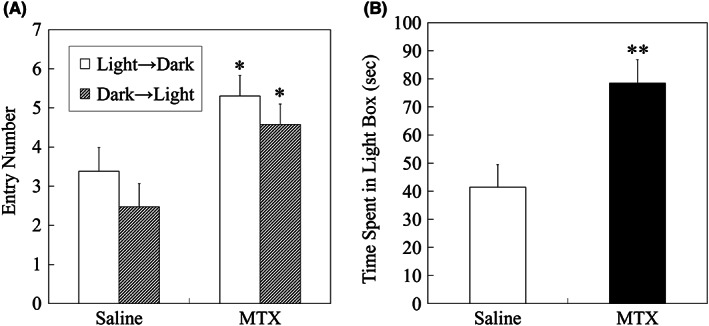
Results of the light and dark box test. (A) Entries to the light and the dark boxes. Significantly higher entry numbers to both the light and the dark boxes were confirmed in MTX group compared to saline group, analyzed by a two‐tailed t‐test (*: *P* < 0.05). (B) Time spent in the light box. MTX group rats spent significantly longer time in the light box than saline group, analyzed by a two‐tailed t‐test (**: *P* < 0.01). Data are expressed as mean ± SEM. The number of animals was n = 21 and n = 26 for saline group and MTX group, respectively

In the LD test, MTX‐treated group showed significantly higher entry numbers to both the light and the dark boxes compared to saline‐treated group (*P* = 0.0107 and *P* = 0.0188, respectively; Figure 4A). Time spent in the light box was significantly higher in MTX‐treated group than in saline‐treated group (*P* = 0.0031; Figure 4B).

**FIGURE 5:**
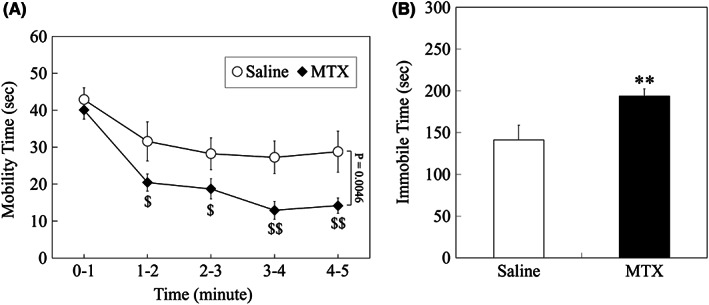
Results of the forced swimming test. (A) Time course of swimming time (mobility time), plotted per minute. There was a significant difference in swimming time between saline‐treated group and MTX‐treated group over the time course, analyzed by a mixed model for repeated measures method. Then, data (group‐by‐time interaction) were analyzed per minute with the closed testing procedure, and significantly less swimming time was confirmed in MTX‐treated group compared to saline‐treated group from 1 minute after placed in water ($: *P* < 0.05, $$: *P* < 0.01). (B) Total immobility time for 5 minutes. MTX group showed significantly longer immobility time than saline group, analyzed by a two‐tailed t‐test (**: *P* < 0.01).Data are expressed as mean ± SEM. The number of animals was n = 11 and n = 21 for saline group and MTX group, respectively

In the FST test, in order to see time course changes in swimming time, data were tallied per minute and difference in overall means between the two groups was compared over the time course using the data by a MMRM method. There was a significant difference in swimming time between saline‐treated group and MTX‐treated group over the time course (*P* = 0.0046; Figure 5A). Therefore, data (group‐by‐time interaction) were then analyzed per minute with the closed testing procedure, and significantly less swimming time was confirmed in MTX‐treated group compared to saline‐treated group from 1 minute after placed in water (0–1 minute: *P* = 0.5482, 1–2 minute: *P* = 0.0199, 2–3 minute: *P* = 0.0459, 3–4 minute: *P* = 0.0030, and 4–5 minute: *P* = 0.0025; Figure 5A). In Addition, immobility time was calculated for the total measurement time (5 minutes). Compared to saline‐treated group, significantly longer immobility time was confirmed in MTX‐treated group (*P* = 0.0046; Figure 5B).

**FIGURE 6:**
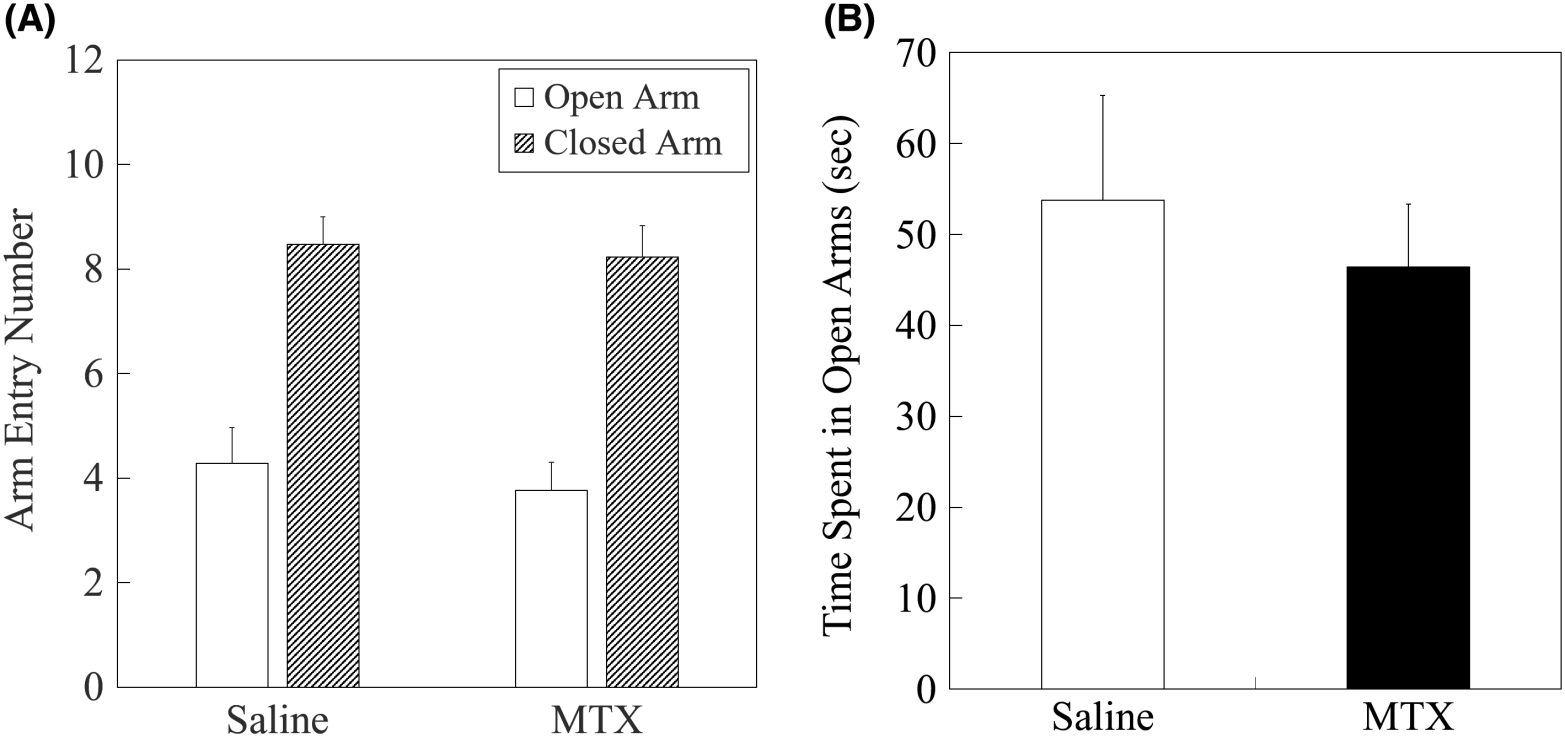
Results of the elevated plus maze test. No significant differences were found in (A) open arm and closed arm entry numbers, and (B) time spent in open arms between saline and MTX groups, analyzed by a two‐tailed t‐test. Data are expressed as mean ± SEM. The number of animals was n = 21 and n = 26 for saline group and MTX group, respectively

In the EPM test, no significant differences in open and closed arm entry numbers (*P* = 0.5482 and *P* = 0.7649, respectively; Figure 6A) and time spent in the open arms (*P* = 0.5720; Figure 6B) were seen between saline‐ and MTX‐treated groups. This may implicate no general anxiety level change in MTX‐treated animals compared to saline‐treated animals.

**FIGURE 7:**
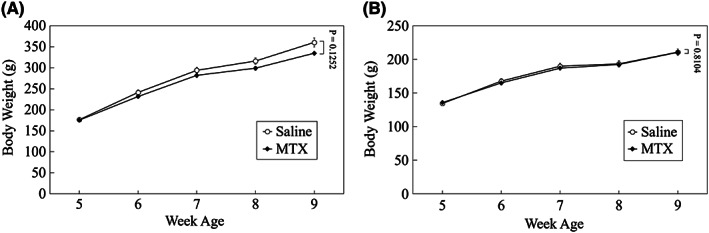
Weekly body weights of saline‐treated rats and MTX‐treated rats. Rat body weights were weekly measured at 5, 6, 7, 8, and 9 weeks old. No significant differences were found in the body weight in (A) male rats (*P* = 0.1252) and (B) female rats (*P* = 0.8104) between saline‐treated and MTX‐treated groups over the time course. Data are expressed as mean ± SEM. The number of male rats: n = 11 and n = 11 for saline group and MTX group, respectively. The number of female rats: n = 10 and n = 15 for saline group and MTX group, respectively

In addition to behavioral tests, weekly body weights (at 5, 6, 7, 8, and 9 weeks old) were compared per gender over the time course between saline‐treated group and MTX‐treated group using a MMRM method. No significant difference was found in the body weight for each gender between the two groups over the time course (*P* = 0.1252 and *P* = 0.8104 for male and female, respectively; Figure 7A,B). For this body weight comparison, the closed testing procedure was not applicable because there was no significant difference in the main effect (group difference over the time course).

## 4 DISCUSSION

Rat offspring treated with MTX, an inhibitor of folate metabolic enzyme DHFR, showed reduction of social interaction. On the other hand, MTX‐treated offspring did not show change in locomotor activity and impairment of PPI. PPI is impaired in patients with schizophrenia and used as a translational paradigm in schizophrenia research in rodents.^31^ Therefore, this animal model may not be associated with schizophrenia, and the primary hypothesis, establishment of a new schizophrenia animal model based on folate metabolic deficiency, was not achieved.

However, MTX‐treated offspring showed increase of immobility time in FST. Importantly, these results are not attributable to animal activity change because MTX‐treated rats did not differ in locomotor activity from saline‐treated rats. Arm entry numbers in the EPM test were not different between saline‐treated group and MTX‐treated group which also implies that MTX‐treated rats had normal motor activity. Moreover, there were no significant body weight changes in both male and female rats between saline‐treated group and MTX‐treated group, indicating that MTX‐treated animals grew normally similar to saline‐treated animals. In addition, we did not record any other observation of clear physical body changes, although anatomical, histological, and biochemical analyses are needed for more understanding of MTX‐treatment’s influence on general health condition of rats. Impairment of social interaction is known as a main feature of ASD.^32^ Social impairments (eg, decreased interest in social interaction) are also seen in MDD patients.^33^ In addition, FST is a popular behavioral test to evaluate depression‐like behavior and antidepressant efficacy in rodents, in which immobility is thought to reflect behavioral despair in rodents.^34^ These, accordingly, suggest that MTX‐treated rat offspring may be exhibiting MDD or ASD related phenotypes. Moreover, MTX‐treated offspring showed increases in time spent in the light box and number of shuttling between the light–dark boxes. These, especially increased shuttling, may indicate changes in anxiety or cognitive levels to environment in this animal model, or phenotypes related to repetitive behaviors seen in ASD patients which is one of core ASD features.^32^ No behavioral changes were, however, found in the EPM test which may imply that the increased shuttling was not a result of changes in anxiety levels. There might also be a different interpretation that the increased immobility time in FST and the increased shuttling in the LD test might be, for example, behaviors associated with catatonia which is seen in psychiatric disorders such as ASD.^35^

To date, associations between folate deficiency and MDD or ASD have been reported by many researchers.^14^, ^36^, ^37^, ^38^, ^39^, ^40^ Importance of neurogenesis has recently been suggested in intervention of MDD.^41^ Although it is unclear if folate deficiency causes inefficient neurogenesis in MDD, deficiency of folate and vitamin B12 has been reported in MDD patients.^10^, ^42^, ^43^ In addition, it has been reported that MDD patients with low serum folate levels were less likely to respond fluoxetine, an antidepressant, compared to patients with normal folate levels.^38^, ^39^ These indicate involvement of folate deficiency in MDD. It has been reported that periconceptional folate deficiency in Wistar rats evoked ASD‐related behaviors in offspring.^44^ Folate supplementation during preconception or pregnancy has been known to reduce risk of offspring to develop ASD in humans.^19^, ^45^, ^46^ Prenatal exposure to valproic acid has been known to cause ASD‐like behaviors (eg, impaired social interaction and repetitive behavior) and neurodevelopmental delay in rat offspring.^47^, ^48^ In this animal model, maternal folate supplementation prevented development of the ASD‐like behaviors in rats prenatally exposed with valproate.^48^ Therefore, folate deficiency during periconception and pregnancy is likely involved in occurrence of ASD in offspring. Furthermore, gene polymorphism in DHFR and MTHFR, folate metabolic enzymes, is reported in patients with ASD.^49^, ^50^ MTX, an anti‐cancer drug, is an analogue of folate and a DHFR inhibitor.^51^, ^52^ It has also been reported that MTX treatment developed characteristics of ASD in children with acute lymphoblastic leukemia who had not been diagnosed with ASD at the time of their cancer diagnoses.^52^ Therefore, folate deficiency and/or folate metabolism deficit seem to play an important role in etiology of psychiatric disorders such as MDD and ASD.

Considering that folate promotes neural stem cell proliferation and neurogenesis^24^, ^25^ and that its supplementation prevents neural tube defects,^22^, ^23^ inhibition of folate metabolism by prenatal MTX treatment may have affected normal CNS development and formation of neural networks in rat fetuses, and subsequently caused behavioral changes related to MDD and/or ASD in MTX‐treated rat offspring. Although still there is a limitation to consider MTX‐treated rats as a MDD or ASD model, and further investigations of this animal model are needed for face validity, construct validity, and predictive validity (eg, conduction of additional behavioral assays, measurement of plasma levels of folate and related molecules, examination of effects on neural stem cell proliferation and neurogenesis, validation for optimal timing and dose selection of MTX treatment, histological and biochemical assays, and gene/protein expression analyses to determine levels of molecules involved in MDD and ASD), at least we can say that prenatal exposure of MTX, an inhibitor of folate metabolic enzyme, affects offspring behaviors.

Finally, this prenatal MTX exposure model may be useful tool to investigate pathology and to evaluate candidate compounds for treatment of MDD and ASD.

## 5 CONCLUSIONS

Our findings at least demonstrated that prenatal MTX treatment caused behavioral changes in rat offspring, which may be associated with MDD and/or ASD, although our primary hypothesis for establishment of an animal model of schizophrenia was not achieved.

## AUTHOR CONTRIBUTIONS

NA conceived and designed the study, performed experiments, analyzed data, wrote the paper. TF reviewed the paper. YK was responsible for statistics. KM conceived and designed the study, performed experiments, analyzed data, reviewed the paper. All authors contributed to finalizing of the paper and had final responsibility for the decision to submit for publication, took part in either drafting and/or revising the paper, and approved the final version of the paper.

## CONFLICT OF INTEREST

All the authors are employees and own stock of Otsuka Pharmaceutical Co., Ltd.

## APPROVAL OF THE RESEARCH PROTOCOL BY AN INSTITUTIONAL REVI EWER BOARD

The experimental procedures in this study were approved and conducted in accordance with Guidelines for Animal Care and Use in Otsuka Pharmaceutical Co, Ltd.

## INFORMED COMSENT

Not applicable.

## REGISTRY AND THE REGISTRATION NO. OF THE STUDY/TRIAL

Not applicable.

## ANIMAL STUDIES

The care and handling of the animals was in accordance with “Guidelines for Animal Care and Use in Otsuka Pharmaceutical Co, Ltd.”

## Data Availability

All data are available in the Supporting Information data files.

## References

[1] Lavreysen H , Langlois X , Donck LV , Nunez JM , Pype S , Lutjens R , et al. Preclinical evaluation of the antipsychotic potential of the mGlu2‐positive allosteric modulator JNJ‐40411813. Pharmacol Res Perspect. 2015;3(2):e00097. 25692027 10.1002/prp2.97 PMC4324682

[2] Monn JA , Massey SM , Valli MJ , Henry SS , Stephenson GA , Bures M , et al. Synthesis and metabotropic glutamate receptor activity of S‐oxidized variants of (−)‐4‐amino‐2‐thiabicyclo‐[3.1.0]hexane‐4,6‐dicarboxylate: identification of potent, selective, and orally bioavailable agonists for mGlu2/3 receptors. J Med Chem. 2007;50(2):233–40. 17228865 10.1021/jm060917u

[3] Boulay D , Pichat P , Dargazanli G , Estenne‐Bouhtou G , Terranova JP , Rogacki N , et al. Characterization of SSR103800, a selective inhibitor of the glycine transporter‐1 in models predictive of therapeutic activity in schizophrenia. Pharmacol Biochem Behav. 2008;91(1):47–58. 18621075 10.1016/j.pbb.2008.06.009

[4] Brown AS , Susser ES . Prenatal nutritional deficiency and risk of adult schizophrenia. Schizophr Bull. 2008;34(6): 1054–63. 18682377 10.1093/schbul/sbn096 PMC2632499

[5] Picker JD , Coyle JT . Do maternal folate and homocysteine levels play a role in neurodevelopmental processes that increase risk for schizophrenia? Harv Rev Psychiatry. 2005;13(4):197–205. 16126606 10.1080/10673220500243372

[6] Wang D , Zhai JX , Liu DW . Serum folate levels in schizophrenia: A meta‐analysis. Psychiatry Res. 2016;235:83–9. 26652840 10.1016/j.psychres.2015.11.045

[7] Cao B , Wang DF , Xu MY , Liu YQ , Yan LL , Wang JY , et al. Lower folate levels in schizophrenia: A meta‐analysis. Psychiatry Res. 2016;245:1–7. 27521746 10.1016/j.psychres.2016.03.003

[8] Goff DC , Bottiglieri T , Arning E , Shih V , Freudenreich O , Evins AE , et al. Folate, homocysteine, and negative symptoms in schizophrenia. Am J Psychiatry. 2004;161(9):1705–8. 15337665 10.1176/appi.ajp.161.9.1705

[9] Roffman JL , Lamberti JS , Achtyes E , Macklin EA , Galendez GC , Raeke LH , et al. Randomized multicenter investigation of folate plus vitamin B12 supplementation in schizophrenia. JAMA Psychiat. 2013;70(5):481–9. 10.1001/jamapsychiatry.2013.900 PMC4394629 23467813

[10] Turksoy N , Bilici R , Yalciner A , Ozdemir YO , Ornek I , Tufan AE , et al. Vitamin B12, folate, and homocysteine levels in patients with obsessive‐compulsive disorder. Neuropsychiatr Dis Treat. 2014;10:1671–5. 25228807 10.2147/NDT.S67668 PMC4164291

[11] Fafouti M , Paparrigopoulos T , Liappas J , Mantouvalos V , Typaldou R , Christodoulou G . Mood disorder with mixed features due to vitamin B(12) and folate deficiency. Gen Hosp Psychiatry. 2002;24(2):106–9. 11869745 10.1016/s0163-8343(01)00181-5

[12] Kale A , Naphade N , Sapkale S , Kamaraju M , Pillai A , Joshi S , et al. Reduced folic acid, vitamin B12 and docosahexaenoic acid and increased homocysteine and cortisol in never‐medicated schizophrenia patients: implications for altered one‐carbon metabolism. Psychiatry Res. 2010;175(1–2):47–53. 19969375 10.1016/j.psychres.2009.01.013

[13] Ezzaher A , Mouhamed DH , Mechri A , Omezzine A , Neffati F , Douki W , et al. Hyperhomocysteinemia in Tunisian bipolar I patients. Psychiatry Clin Neurosci. 2011;65(7):664–71. 22176285 10.1111/j.1440-1819.2011.02284.x

[14] Belardo A , Gevi F , Zolla L . The concomitant lower concentrations of vitamins B6, B9 and B12 may cause methylation deficiency in autistic children. J Nutr Biochem. 2019;70:38–46. 31151052 10.1016/j.jnutbio.2019.04.004

[15] Ramaekers VT , Thony B , Sequeira JM , Ansseau M , Philippe P , Boemer F , et al. Folinic acid treatment for schizophrenia associated with folate receptor autoantibodies. Mol Genet Metab. 2014;113(4):307–14. 25456743 10.1016/j.ymgme.2014.10.002

[16] Frye RE , Sequeira JM , Quadros EV , James SJ , Rossignol DA . Cerebral folate receptor autoantibodies in autism spectrum disorder. Mol Psychiatry. 2013;18(3):369–81. 22230883 10.1038/mp.2011.175 PMC3578948

[17] Crider KS , Yang TP , Berry RJ , Bailey LB . Folate and DNA methylation: a review of molecular mechanisms and the evidence for folate’s role. Adv Nutr. 2012;3(1):21–38. 22332098 10.3945/an.111.000992 PMC3262611

[18] Nazki FH , Sameer AS , Ganaie BA . Folate: metabolism, genes, polymorphisms and the associated diseases. Gene. 2014;533(1):11–20. 24091066 10.1016/j.gene.2013.09.063

[19] Liu X , Zou M , Sun C , Wu L , Chen WX . Prenatal folic acid supplements and offspring’s autism spectrum disorder: a meta‐analysis and meta‐regression. J Autism Dev Disord. 2021;52:522–39. 33743119 10.1007/s10803-021-04951-8 PMC8813730

[20] Shane B . Folate and vitamin B12 metabolism: overview and interaction with riboflavin, vitamin B6, and polymorphisms. Food Nutr Bull. 2008;29(2 Suppl):S5–16; discussion S7‐9. 18709878 10.1177/15648265080292S103

[21] Enko D , Meinitzer A , Brandmayr W , Halwachs‐Baumann G , Schnedl WJ , Kriegshauser G . Association between increased plasma levels of homocysteine and depression observed in individuals with primary lactose malabsorption. PLoS One. 2018;13(8):e0202567. 30138390 10.1371/journal.pone.0202567 PMC6107192

[22] Czeizel AE , Dudas I . Prevention of the first occurrence of neural‐tube defects by periconceptional vitamin supplementation. N Engl J Med. 1992;327(26):1832–5. 1307234 10.1056/NEJM199212243272602

[23] Padmanabhan R , Shafiullah MM . Amelioration of sodium valproate‐induced neural tube defects in mouse fetuses by maternal folic acid supplementation during gestation. Congenit Anom (Kyoto). 2003;43(1):29–40. 12692401 10.1111/j.1741-4520.2003.tb01024.x

[24] Ichi S , Costa FF , Bischof JM , Nakazaki H , Shen YW , Boshnjaku V , et al. Folic acid remodels chromatin on Hes1 and Neurog2 promoters during caudal neural tube development. J Biol Chem. 2010;285(47):36922–32. 20833714 10.1074/jbc.M110.126714 PMC2978621

[25] Wang X , Li W , Li Z , Ma Y , Yan J , Wilson JX , et al. Maternal folic acid supplementation during pregnancy promotes neurogenesis and synaptogenesis in neonatal rat offspring. Cereb Cortex. 2019;29(8):3390–7. 30137237 10.1093/cercor/bhy207

[26] Rice D , Barone S Jr . Critical periods of vulnerability for the developing nervous system: evidence from humans and animal models. Environ Health Perspect. 2000;108(Suppl 3):511–33. 10852851 10.1289/ehp.00108s3511 PMC1637807

[27] Futamura T , Kakita A , Tohmi M , Sotoyama H , Takahashi H , Nawa H . Neonatal perturbation of neurotrophic signaling results in abnormal sensorimotor gating and social interaction in adults: implication for epidermal growth factor in cognitive development. Mol Psychiatry. 2003;8(1):19–29. 12556905 10.1038/sj.mp.4001138

[28] Mori A , Ohashi S , Nakai M , Moriizumi T , Mitsumoto Y . Neural mechanisms underlying motor dysfunction as detected by the tail suspension test in MPTP‐treated C57BL/6 mice. Neurosci Res. 2005;51(3):265–74. 15710490 10.1016/j.neures.2004.11.008

[29] Tottori K , Miwa T , Uwahodo Y , Yamada S , Nakai M , Oshiro Y , et al. Antidepressant‐like responses to the combined sigma and 5‐HT1A receptor agonist OPC‐14523. Neuropharmacology. 2001;41(8):976–88. 11747902 10.1016/s0028-3908(01)00147-2

[30] Li G , Taljaard M , Van den Heuvel ER , Levine MA , Cook DJ , Wells GA , et al. An introduction to multiplicity issues in clinical trials: the what, why, when and how. Int J Epidemiol. 2017;46(2):746–55. 28025257 10.1093/ije/dyw320

[31] Yee BK , Singer P . A conceptual and practical guide to the behavioural evaluation of animal models of the symptomatology and therapy of schizophrenia. Cell Tissue Res. 2013;354(1):221–46. 23579553 10.1007/s00441-013-1611-0 PMC3791321

[32] Tartaglione AM , Schiavi S , Calamandrei G , Trezza V . Prenatal valproate in rodents as a tool to understand the neural underpinnings of social dysfunctions in autism spectrum disorder. Neuropharmacology. 2019;159:107477. 30639388 10.1016/j.neuropharm.2018.12.024

[33] Kupferberg A , Bicks L , Hasler G . Social functioning in major depressive disorder. Neurosci Biobehav Rev. 2016;69:313–32. 27395342 10.1016/j.neubiorev.2016.07.002

[34] Pollak DD , Rey CE , Monje FJ . Rodent models in depression research: classical strategies and new directions. Ann Med. 2010;42(4):252–64. 20367120 10.3109/07853891003769957

[35] Mazzone L , Postorino V , Valeri G , Vicari S . Catatonia in patients with autism: prevalence and management. CNS Drugs. 2014;28(3):205–15. 24504828 10.1007/s40263-014-0143-9

[36] Abou‐Saleh MT , Coppen A . Serum and red blood cell folate in depression. Acta Psychiatr Scand. 1989;80(1):78–82. 2763862 10.1111/j.1600-0447.1989.tb01303.x

[37] Coppen A , Swade C , Jones SA , Armstrong RA , Blair JA , Leeming RJ . Depression and tetrahydrobiopterin: the folate connection. J Affect Disord. 1989;16(2–3):103–7. 2522108 10.1016/0165-0327(89)90062-1

[38] Fava M , Borus JS , Alpert JE , Nierenberg AA , Rosenbaum JF , Bottiglieri T . Folate, vitamin B12, and homocysteine in major depressive disorder. Am J Psychiatry. 1997;154(3):426–8. 9054796 10.1176/ajp.154.3.426

[39] Papakostas GI , Petersen T , Lebowitz BD , Mischoulon D , Ryan JL , Nierenberg AA , et al. The relationship between serum folate, vitamin B12, and homocysteine levels in major depressive disorder and the timing of improvement with fluoxetine. Int J Neuropsychopharmacol. 2005;8(4):523–8. 15877935 10.1017/S1461145705005195

[40] Frye RE , Rossignol DA , Scahill L , McDougle CJ , Huberman H , Quadros EV . Treatment of Folate Metabolism Abnormalities in Autism Spectrum Disorder. Semin Pediatr Neurol. 2020;35:100835. 32892962 10.1016/j.spen.2020.100835 PMC7477301

[41] Warner‐Schmidt JL , Duman RS . Hippocampal neurogenesis: opposing effects of stress and antidepressant treatment. Hippocampus. 2006;16(3):239–49. 16425236 10.1002/hipo.20156

[42] Bottiglieri T , Laundy M , Crellin R , Toone BK , Carney MW , Reynolds EH . Homocysteine, folate, methylation, and monoamine metabolism in depression. J Neurol Neurosurg Psychiatry. 2000;69(2):228–32. 10896698 10.1136/jnnp.69.2.228 PMC1737050

[43] Kim JM , Stewart R , Kim SW , Yang SJ , Shin IS , Yoon JS . Predictive value of folate, vitamin B12 and homocysteine levels in late‐life depression. Br J Psychiatry. 2008;192(4):268–74. 18378986 10.1192/bjp.bp.107.039511

[44] Degroote S , Hunting D , Takser L . Periconceptional folate deficiency leads to autism‐like traits in Wistar rat offspring. Neurotoxicol Teratol. 2018;66:132–8. 29305196 10.1016/j.ntt.2017.12.008

[45] Gao Y , Sheng C , Xie RH , Sun W , Asztalos E , Moddemann D , et al. New perspective on impact of folic acid supplementation during pregnancy on neurodevelopment/autism in the offspring children ‐ a systematic review. PLoS One. 2016;11(11):e0165626. 27875541 10.1371/journal.pone.0165626 PMC5119728

[46] Suren P , Roth C , Bresnahan M , Haugen M , Hornig M , Hirtz D , et al. Association between maternal use of folic acid supplements and risk of autism spectrum disorders in children. JAMA. 2013;309(6):570–7. 23403681 10.1001/jama.2012.155925 PMC3908544

[47] Elnahas EM , Abuelezz SA , Mohamad MI , Nabil MM , Abdelraouf SM , Bahaa N , et al. Validation of prenatal versus postnatal valproic acid rat models of autism: A behavioral and neurobiological study. Prog Neuropsychopharmacol Biol Psychiatry. 2021;108:110185. 33238165 10.1016/j.pnpbp.2020.110185

[48] Di Y , Li Z , Li J , Cheng Q , Zheng Q , Zhai C , et al. Maternal folic acid supplementation prevents autistic behaviors in a rat model induced by prenatal exposure to valproic acid. Food Funct. 2021;12(10):4544–55. 33903876 10.1039/d0fo02926b

[49] Adams M , Lucock M , Stuart J , Fardell S , Baker K , Ng X . Preliminary evidence for involvement of the folate gene polymorphism 19bp deletion‐DHFR in occurrence of autism. Neurosci Lett. 2007;422(1):24–9. 17597297 10.1016/j.neulet.2007.05.025

[50] Li Y , Qiu S , Shi J , Guo Y , Li Z , Cheng Y , et al. Association between MTHFR C677T/A1298C and susceptibility to autism spectrum disorders: a meta‐analysis. BMC Pediatr. 2020;20(1):449. 32972375 10.1186/s12887-020-02330-3 PMC7517654

[51] Bertino JR . Cancer research: from folate antagonism to molecular targets. Best Pract Res Clin Haematol. 2009;22(4):577–82. 19959110 10.1016/j.beha.2009.09.004

[52] Kamen BA , Chukoskie L . Autism Speaks: meeting on folate metabolism and Autism spectrum disorders, March 19‐20, 2009, Washington. DC J Pediatr Hematol Oncol. 2011;33(3):208–15. 21427559 10.1097/MPH.0b013e31820ff78e

